# Network meta-analysis of comparative efficacy and safety of intubation devices in children

**DOI:** 10.1038/s41598-023-45173-5

**Published:** 2023-10-30

**Authors:** Yu Ming, Shujuan Chu, Kai Yang, Zhao Zhang, Zhouyang Wu

**Affiliations:** 1 College of Medicine and Health Science, Wuhan Polytechnic University, Wuhan, 430023 Hubei China; 2 Department of Anesthesiology, Union Hospital, Tongji Medical College, Huazhong University of Science and Technology, Wuhan, 430022 China

**Keywords:** Diseases, Medical research

## Abstract

To evaluate the comparative efficacy and safety of different intubation devices on intubation outcomes in pediatric intubation. We identified relevant studies from previous meta-analyses and literature retrieval in PubMed, EMBASE, and Cochrane Library. The primary outcome was the first-pass success (FPS), and the secondary outcome included the time to intubation (TTI) and the risk of local complications (LC). Network meta-analysis was performed using STATA 14.0. Twenty-three randomized comparative trials (RCTs) including 12 devices were included. Compared with Macintosh, Airtraq (odds ratio [OR] = 13.05, 95% confidence interval [CI] = 4.68 to 36.38), Miller (OR = 4.77, 95%CI = 1.32 to 17.22), Glidescope (OR = 2.76, 95%CrI = 1.60 to 4.75) and McGrath (OR = 4.61, 95%CI = 1.18 to 17.99) obtained higher PFS. Meanwhile, Airtraq was superior to Glidescope (OR = 0.21, 95%CI = 0.07 to 0.65) for PFS. For TTI, Canada was superior to other intubation devices, as well as CMAC was superior to TruViewEVO2, Glidescope, and StorzDCI. Airtraq lowered the risk of LC compared with Macintosh and Pentax but there was no statistical difference between Airtraq and KingVision. Airtraq may be the optimal option for FPS, Canada for TTI, and KingVision for LC in pediatric intubation.

## Introduction

Endotracheal intubation maintains airway patency in children with or without a difficult airway and protects the airway in patients with altered mental status^1^. The pediatric airway differs distinctively from the adult airway in both anatomy and physiology^2^, which include thinner and tender trachea, weaker respiratory muscles, incomplete bone growth, poor intubation tolerance, and increased oxygen demand with low oxygen reserves^3^, making pediatric intubation a great challenge. Consequently, multiple attempts at intubation in children can lead to complications such as the inability to ventilate or intubate leading to hypoxia, pharyngeal and laryngeal injuries, and airway oedema^4^.

Prevention of complications associated with intubation in children is critical, and various intubation strategies have been proposed to prevent complications, such as minimizing the number of direct laryngoscopy attempts^5^ and early conversion to videolaryngoscopy^6^. Notably, videolaryngoscopy is increasingly being used for intubation in children, providing adequate glottis view regardless of airway difficulty^7,8^. In addition, videolaryngoscopy provides instructors the opportunity to observe and proctor students' intubation techniques to maximize their learning curve and success rate^9,10^. To date, several randomized controlled trials (RCTs) have investigated the comparative efficacy and safety of various intubation devices in children^11–14^.

A recent traditional pair-wise meta-analysis showed that while videolaryngoscopes significantly reduced intubation time^1^, there is no advantage for first pass success (FPS), but it was challenging to identify the optimal intubation device in the absence of a meta-analysis to distinguish these devices in terms of applicability in a specific setting. As an expansion of traditional pair-wise meta-analysis, the development of network meta-analysis allows for the simultaneous comparison of multiple treatments^15^. Network meta-analysis enables a comprehensive comparison of data from two or more eligible studies by combining direct and indirect evidence^15^. We, therefore, conducted this network meta-analysis to consistently rank the efficacy and safety of different intubation devices for intubation in children based on all the available evidence.

## Material and methods

The present network meta-analysis was reported in accordance with the Preferred Reporting Items for Systematic Reviews and Meta-Analyses (PRISMA) extension statement for reporting systematic reviews incorporating network meta-analysis (PRISMA-NMA)^16,17^. This systematic review and meta-analysis is registered on PROSPERO with the registration number CRD42023423746.

### Information sources

We first identified randomized comparative trials (RCTs) from a previous meta-analysis^1^ investigating the comparative efficacy and safety of different intubation devices in pediatric intubation. Two independent authors then searched PubMed, Embase, and the Cochrane Library on 20 November 2021. The detailed search query is shown in Table S1. We also checked the reference lists of eligible studies to add additional relevant studies. Any disagreements were resolved by consulting a third author.

### Selection criteria

Eligible studies were identified by screening the titles, abstracts, and full texts with EndNote software version X9. We designed the following inclusion criteria based on the previous meta-analysis:

1. RCTs compared two or more intubation devices in anesthetized pediatric patients (aged ≥ 12 months and < 18 years);
2. reported first-pass success rate (FPS), time to intubation (TTI), or the incidence of local complications;

Studies were excluded if they met one of the following criteria:

1. manikin study;
2. Patients were neonates and infants;
3. no data available;
4. duplicate studies.

### Data extraction

Two independent authors extracted the following data: first author, origin, publication year, American Society of Anaesthesiologists (ASA) physical status, sample size, the proportion of male patients, age, weight, and device used for videolaryngoscopy and direct laryngoscopy, and outcomes. Moreover, detailed information on the risk of bias was extracted as requested by the Cochrane risk of bias tool^18^. Any disagreements were resolved by consulting a third author for consensus.

### Data items

In this network meta-analysis, we defined FPS as the primary outcome and TTI and LC as secondary outcomes. LC was defined to include dental or lip trauma, minor bleeding, or mucosal injury^19^. For the continuous variable (i.e., TTI), we used the recommended formula to estimate the sample mean and standard deviation where data was not available in this format^20^. When the underlying data were not available, we contacted the leading author for more information.

### Geometry of the network

We generated a conventional network graph to explore the configuration of the network for a single outcome. A node represented an intubation device, and a line between the two nodes indicated that the two intubation devices were directly comparable. Furthermore, the size of a node represented the number of patients, and the thickness of a line represented the number of direct comparisons.

### Risk of bias within the study

Two independent authors assessed the methodological quality of included studies using the Cochrane Collaboration tool, Risk of Bias 2 tool (RoB2) (The Cochrane Collaboration, Oxford, UK)^18^. Individual studies were assessed on the following seven items: random sequence generation, allocation concealment, blinding of participants and personnel, blinding of outcome assessment, incomplete outcome data, selective outcome reporting, and other sources. Each item was classified as low, unclear, or high based on how well the actual information matched the assessment criteria. Any disagreements between the authors were resolved by discussion or consultation with a third reviewer.

### Risk of bias across studies

We used funnel plots to test if reporting bias, heterogeneity, methodological quality, or chance interfered with the final results^21^. Plots were generated for the primary and secondary outcomes.

### Statistical analysis

Random-effect frequentist network meta-analyses were performed using STATA software (version 14; StataCorp LP, College Station, Texas, USA) with the "network" command. Odds ratios (ORs) with 95% confidence intervals in (CIs) were calculated as effect sizes. Meanwhile, we used graphical tools developed by Chaimani and colleagues^22^ to visually represent the results.

We first appraised the plausibility of the transitivity hypothesis based on the design characteristics and methods of the studies included in this network meta-analysis^23^. We examined overall consistency using the design-by-treatment interaction model^24,25^, and we also used the method described by Lu and Ades^26^ to assess loop inconsistency. We used the side-splitting model to check the inconsistency between the direct and indirect evidence^27^.

We calculated the surface under the cumulative ranking (SUCRA) line to determine how different intubation devices rank in terms of individual outcomes. The higher the SUCRA value, the greater the probability t of a higher rank^28^. Comparison-adjusted funnel plots for individual outcomes were generated^29^.

## Results

### Study selection

The process of study retrieval and selection is shown in Fig. S1. A total of 109 studies were captured following an updated literature retrieval in PubMed, Embase, and the Cochrane Library. After the removal of duplicate studies and a careful review of titles and abstracts, four studies^11–14^ were considered to meet our inclusion criteria. For full-text evaluation, we excluded five studies due to manikin studies (n = 3), abstract (n = 1), and insufficient information about direct intubation devices (n = 1). In addition, we identified 19 eligible studies^30–46^ from previous meta-analysis. Finally, 23 studies published between 2008 to 2021 were included in the present network meta-analysis^11–14, 30–48^.

### Basic characteristics of included studies

**Figure 1:**
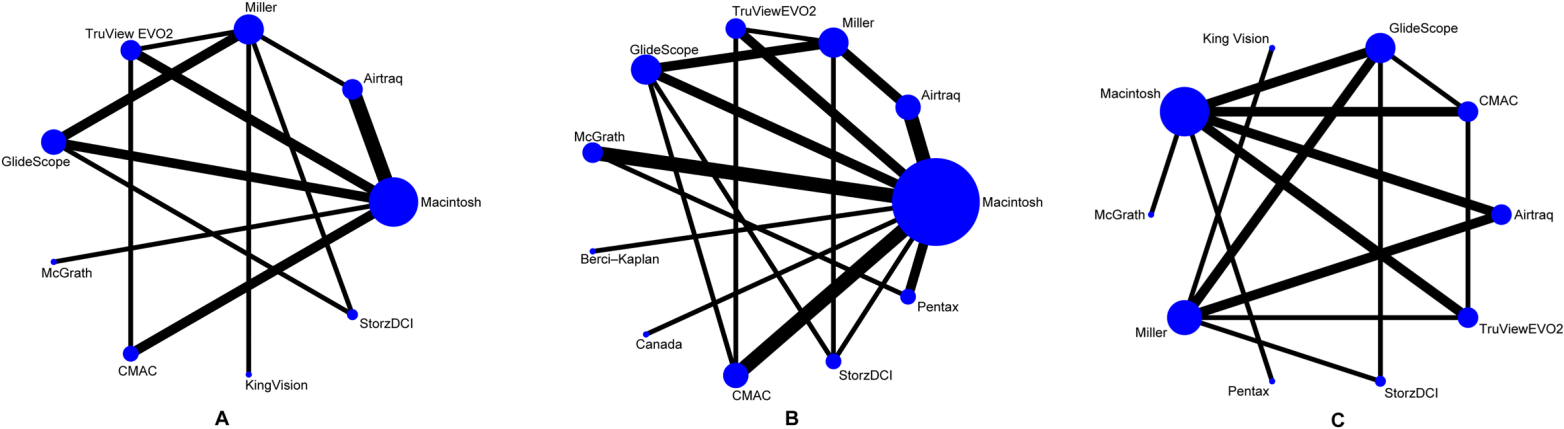
Network plot for first-pass success (**A**), time to intubation (**B**), and local complications (**C**). The size of each node represents the number of patients included in studies featuring that device. The thickness of the lines connecting the nodes is proportional to the number of head-to-head studies in each comparison.

Table 1 documented the basic characteristics of the included studies. Among 23 studies, 1873 children were enrolled in our network meta-analysis. Six studies^14,32, 33, 36, 44, 45^ reported details of the ASA status of eligible patients. A total of 12 intubation devices were identified, including Macintosh, Miller, GlideScope (hyperangulated), Pentax (channeled), CMAC (Macintosh-style), TruviewEVO2 (hyperangulated), Airtraq (hyperangulated), KingVision (hyperangulated), StorzDCI, McGrath MAC (Macintosh-style), and Berci–Kaplan. Figure 1 shows the structure of the evidence for all results categorised by specific device name, and Fig. S5 shows the netplot categorised by device type.

**Table 1:** Basic characteristics of the included studies.

Study	Origin	ASA score	Intervention arms	Sample size	Male, %	Age, years	Weight, kg	Outcomes
Ali et al.^30^	India	1–2	Airtraq	17	NA	2.9 ± 1.5	11.9 ± 1.3	FPS, TTI, LC
			Macintosh	17	NA	2.7 ± 1.4	11.6 ± 1.2	
Das et al.^31^	India	1–2	Airtraq	30	83.3	6.15 ± 2.64	16.5 ± 3.87	FPS, TTI, LC
			Miller	30	63.3	5.4 ± 1.78	15.9 ± 2.53	
Inal et al.^32^	Turkey	NA	TruView EVO2	25	56.0	4.68 ± 1.7	16.84 ± 4.52	FPS, TTI, LC
			Miller	25	60.0	4.72 ± 1.5	16.88 ± 4.25	
Kim et al.^36^	Korea	NA	GlideScope	100	63.1	6.5 ± 4.2	27.3 ± 16.2	FPS, TTI
			Macintosh	103	57.0	6.1 ± 3.8	24.4 ± 11.9	
Kim et al.^34^	Korea	1–2	GlideScope	40	52.5	5.1 ± 2.0	21.4 ± 7.9	FPS, TTI, LC
			Miller	40	42.5	5.5 ± 1.9	20.2 ± 5.3	
Kim et al.^35^	Korea	1–2	McGrath	42	NA	NA	NA	FPS, TTI, LC
			Macintosh	42	NA	NA	NA	
Macnair et al.^37^	UK	1–2	Berci–Kaplan	30	NA	8.9 ± 3.6	34.9 ± 16.0	TTI
			Macintosh	30	NA			
Orozco et al.^38^	Venezuela	1–2	Airtraq	40	52.5	5 ± 2	17 ± 4	FPS, TTI, LC
			Macintosh	40	57.5	4 ± 2	18 ± 4	
Pangasa et al.^39^	India	1–2	TruView EVO2	25	56.0	4.5 ± 2.1	14.7 ± 5.0	FPS, TTI, LC
			Macintosh	25	64.0	5.4 ± 2.1	16.1 ± 4.9	
Redel et al.^40^	Germany	1–3	GlideScope	30	40.0	4.4 ± 2.08	19 ± 8	TTI, LC
			Macintosh	30	63.0	4.17 ± 2.08	17 ± 5	
Riad et al.^41^	Canada	1	Canada	25	52.0	5.76 ± 2.2	23.98 ± 11.7	TTI
			Macintosh	25	56.0	5.88 ± 2.6	23.98 ± 11.7	
Singh et al.^42^	India	1–2	TruView EVO2	50	68.0	3.8 ± 2.0	14.7 ± 3.9	FPS, TTI, LC
			Macintosh	50	84.0	3.9 ± 1.8	14.7 ± 3.9	
			CMAC	50	68.0	3.5 ± 1.8	14.7 ± 3.9	
Vlatten et al.^44^	Canada	NA	Airtraq	24	75.0	3.25 ± 0.77	14 ± 1.5	FPS, TTI
			Macintosh	25	68.0	3.33 ± 0.76	13 ± 1.25	
Vlatten et al.^45^	Canada	NA	Storz DCI	28	60.7	2.67 ± 0.54	14.3 ± 1.6	TTI
			Macintosh	28	75.0	2.08 ± 0.46	14.6 ± 1.75	
White et al.^46^	UK	1–2	Airtraq	20	NA	1.6 ± 0.85	11.8 ± 1.33	TTI, LC
			Miller	20	NA	3.3 ± 0.6	14.9 ± 1.2	
Yi et al.^47^	Korea	1–2	Pentax	68	68.0	5 ± 1.48	21 ± 6	TTI, LC
			Macintosh	68	63.0	5 ± 2.2	20 ± 6	
Yoo et al.^48^	Korea	1–2	McGrath	36	77.1	7 ± 1.48	25 ± 3	TTI
			Macintosh	35	77.8	7 ± 1.48	25 ± 2	
			Pentax	36	71.4	7 ± 2.2	25 ± 3.25	
Jagannathan et al.^33^	USA	NA	King Vision	100	84.0	0.75 ± 0.18	9.2 ± 0.73	FPS, LC
			Miller	100	84.0	0.88 ± 0.18	9 ± 0.65	
Vadi et al.^43^	USA	1–3	GlideScope	31	83.3	0.56 ± 0.18	8 ± 0.43	FPS, TTI, LC
			Miller	31	63.3	0.74 ± 0.29	8.5 ± 0.45	
			Storz DCI	31	67.7	0.65 ± 0.23	8.4 ± 0.5	
Hajiyeva et al.^11^	Turkey	1–3	CMAC	28	NA	5.86 ± 3.41	22.89 ± 9.29	FPS, TTI, LC
			Macintosh	28	NA	5.75 ± 3.62	19.79 ± 7.89	
Srinivasan et al.^12^	India	1–2	McGrath	30	53.3	1.42 ± 1.16	8.30 ± 1.98	TTI
			Macintosh	30	46.7	1.49 ± 1.08	8.75 ± 2.58	
Teo et al.^13^	Malaysia	1–2	GlideScope	32	53.1	6.81 ± 2.86	20.13 ± 8.14	TTI, LC
			CMAC	33	57.6	6.06 ± 2.03	17.05 ± 3.94	
Zabani et al.^14^	Saudi Arabia	NA	GlideScope	25	NA	29.3 ± 37.0	NA	FPS, TTI, LC
			Macintosh	25	NA	25.2 ± 30.6	NA	

FPS, first-pass success; TTI, time to intubation; LC, local complications; ASA, American Society of Anesthesiologists; NA, not applicable.

### Risk of bias within studies

**Figure 2:**
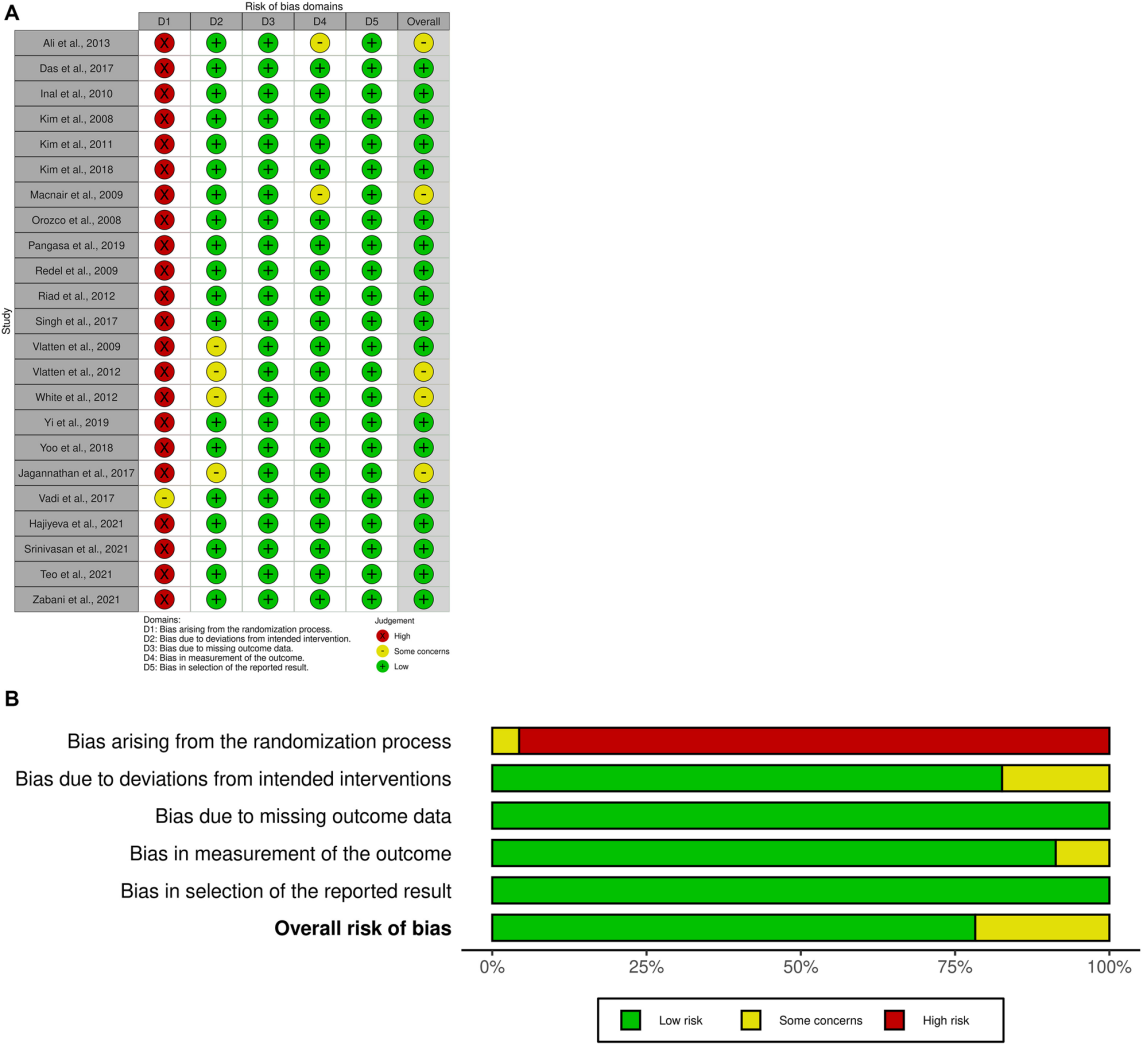
Risk of bias assessments. y, u, and n indicate low, unclear, and high risk of bias, respectively.

Of the 23 included studies, 20 studies^12–14, 30–39, 41–44, 46, 47, 49^ explicitly reported methods for generating random sequence, and 13 studies^13,31, 32, 34, 35, 37–40, 42, 43, 47, 48^ described methods for performing allocation concealment. Most studies^12–14, 30–43^ did not blind participants and personnel. Two studies^31,37^ made this explicit to blind outcome assessors, but the remaining studies did not report details. The 'incomplete outcome data' and 'selective reporting' categories showed a low risk of bias for all included studies. Most studies had low risk in other sources. The details of the risk of bias are displayed in Fig. 2.

### Risk of bias across studies

We generated comparison-adjusted funnel plots for all outcomes. Symmetric funnel plots indicated no bias is present for FPS and LC. However, an asymmetric funnel plot for TTI suggested a potential bias. See Fig. S2 for a funnel plot categorised by device name and Fig. S6 for a funnel plot categorised by actual type of device.

### Synthesis of results

#### Classified by device type

As shown in Fig. S7, we provide estimated pairwise pooled effects as well as 95% confidence intervals (CIs) for the results. We chose the consistency model to calculate all results as the consistency model tests for FPS (chi2 = 0.17, P = 0.667), TTI (chi2 = 0.67, P = 0.715), and LC (chi2 = 0.01, P = 0.924) showed consistent results.

**Figure 3:**
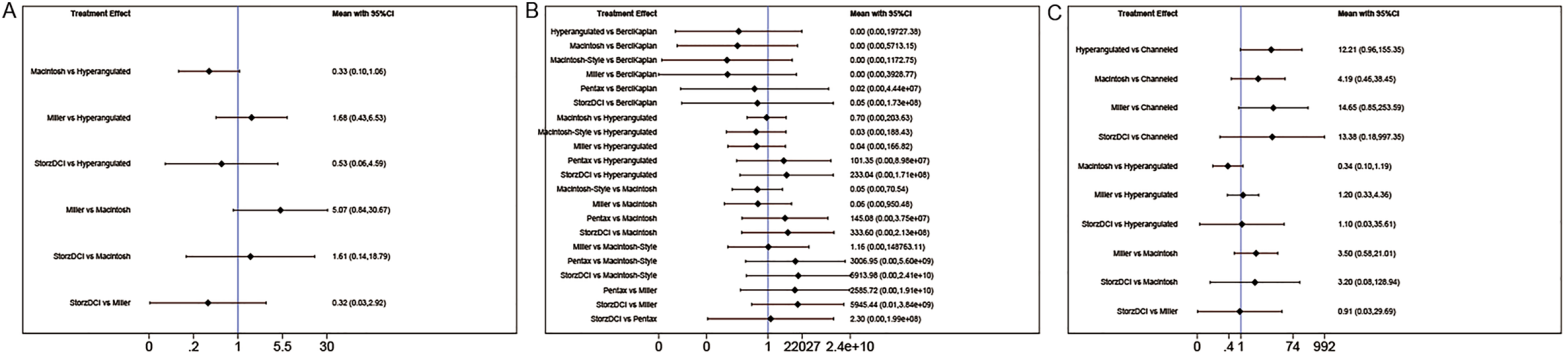
Forest of relative effects of different devices in terms of first-pass success (**A**), time to intubation (**B**), and local complications (**C**), Classified by type of laryngoscope.

According to the network meta-analysis, FPS, TTI, and LC parameters did not vary significantly between devices. Figure 3 is a forest plot of the meta-analysis as a whole, showing the relative impact of several intubation device types. Figure S8 shows the SUCRA plots for each intubation device at different outcomes.

#### Classified by device name

We presented estimated pair-wise summary effects for outcomes, showing a 95% confidence interval (CI), as shown in Fig. S3. We selected the consistency model to calculate all results because consistency model tests for FPS (chi2 = 5.33, P = 0.377), TTI (chi2 = 2.31, P = 0.986), and LC (chi2 = 0.40, P = 0.999) showed agreement.

**Figure 4:**
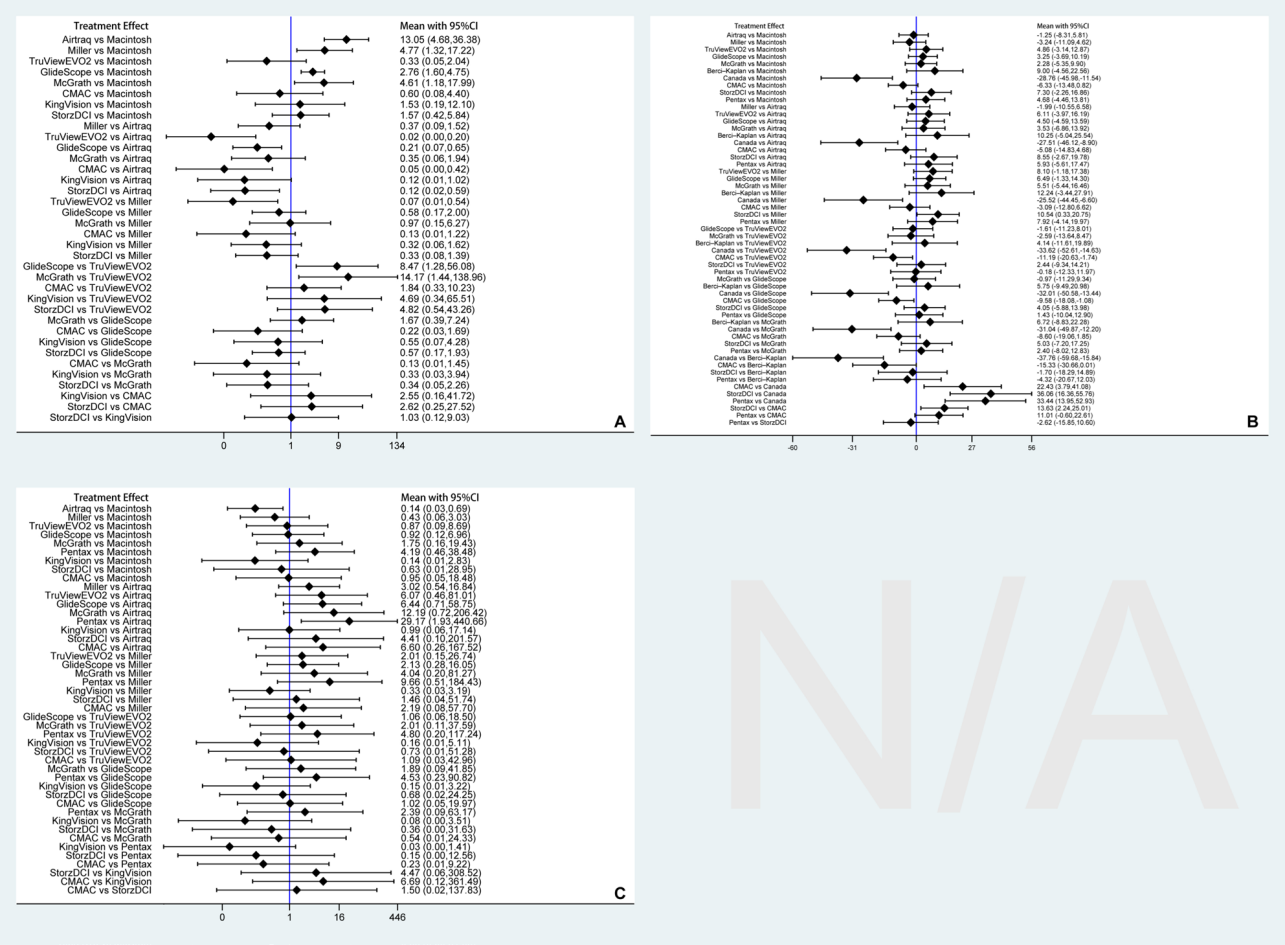
Forest of relative effects of different devices in terms of first-pass success (**A**), time to intubation (**B**), and local complications (**C**).

Among 23 studies, 14 studies^10,14, 30–36, 38, 39, 42–44^ involving 1239 patients reported the data of FPS for 9 intubation devices. A network meta-analysis suggested that compared with Macintosh device, Airtraq (OR = 13.05, 95%CI = 4.68 to 36.38), Miller (OR = 4.77, 95%CI = 1.32 to 17.22), GlideScope (OR = 2.76, 95%CI = 1.60 to 4.75), and McGrath (OR = 4.61, 95%CI = 1.18 to 17.99) significantly increased the FPS. Meanwhile, the Airtraq device was also superior to GlideScope (OR = 0.21, 95%CI = 0.07 to 0.65), CMAC (OR = 0.05; 95%CrI = 0.00 to 0.42), and StorzDCI (OR = 0.12, 95%CI = 0.02 to 0.59). Ranking probabilities suggested that Airtraq had the highest probability of ranking first (80.7%), followed by Miller (37.5%) and McGrath (23.3%). The relative effects of different intubation devices for FPS are presented in Fig. 4A. The SUCRA plots of each intubation device regarding FPS are shown in Fig. S4A.

A total of 22 studies^11–14, 30–32, 34–43^, involving 1673 patients, reported the data of TTI for 11 intubation devices. Network meta-analysis suggested that the Canada intubation device significantly reduced the TTI compared with Macintosh (MD = − 28.76, 95%CI = − 45.98 to − 11.54), Airtraq (MD = − 27.51, 95%CI = − 46.12 to − 8.90), Miller (MD = − 25.52, 95%CI = − 44.45 to − 6.60), TruViewEVO2 (MD = − 33.62, 95%CI = − 52.61 to − 14.63), glideScope (MD = − 32.01, 95%CI = − 50.58 to − 13.44), McGrath (MD = − 31.04, 95%CI = − 49.87 to − 12.20), Berci-Kaplan (MD = − 37.76, 95%CrI = − 59.68 to − 15.84), CMAC (MD = 22.43, 95%CI = 3.79 to 41.08), StorzDCI (MD = 36.06, 95%CrI = 16.36 to 55.76), and Pentax (MD = 33.44, 95%CI = 13.95 to 52.93). Meanwhile, CMAC is superior to TruViewEVO2 (MD = − 11.19, 95%CI = − 20.63 to − 1.74), GlideScope (MD = − 9.58, 95%CI = − 18.08 to − 1.08), and StorzDCI (MD = 13.63, 95%CI = 2.24 to 25.01). Ranking probabilities suggested that Canada had the highest probability of ranking first (98.8%), followed by CMAC (64.1%) and Miller (34.6%). The relative effects of different intubation devices for FPS are presented in Fig. 4B. The SUCRA plots of each intubation device regarding FPS are shown in Fig. S4B.

Among the included studies, a total of 16 studies^11,13, 14, 30–35, 38–40, 42, 43, 46, 47^, involving 1288 patients, reported the data of LC for 10 intubation devices. Network meta-analysis suggested that Airtraq significantly reduced the risk of LC compared with Macintosh (OR = 0.14, 95%CI = 0.03 to 0.69) and Pentax (OR = 29.17, 95%CI = 1.93 to 440.66). The remaining comparisons did not reach statistical significance. Ranking probabilities suggested that KingVision had the highest probability of ranking first (39.5%), followed by Airtraq (35.7%) and Miller (24.7%). The relative effects of different intubation devices for FPS are presented in Fig. 4C. The SUCRA plots of each intubation device regarding FPS are shown in Fig. S4C.

### Inconsistency examination

Node-splitting method was used to examine loop inconsistency in our network meta-analysis. The direct effects were found to be inconsistent with indirect effects in terms of FPS and LC. However, we found that the direct effect was inconsistent with the indirect effect in terms of TTI. All results of inconsistency examinations are shown in Table S2.

## Discussion

There is no consensus on the optimal device for pediatric endotracheal intubation due to the anatomical and physiological differences between the airways of pediatric and adult patients^2^. A previous meta-analysis^1^ showed that, compared with direct laryngoscopy intubation, intubation with videolaryngoscopy devices did not significantly reduce first attempt failure and lower TTI. However, many intubation devices are available for videolaryngoscopy, it is unclear which devices should be preferred in the specific setting of pediatric patients. We therefore performed this network meta-analysis to compare the relative performance of different intubation devices for intubation in children.

Our network meta-analysis reported several important findings as follows: (1) Airtraq, GlideScope, and McGrath significantly improved the FPS in pediatic intubation, and Airtraq is better than GlideScope, CMAC, and StorzDCI in the improvement of FPS; (2) Canada is associated with significant reduction of TTI compared with other intubation devices, and CMAC also significantly reduce the TTI compared with TruViewEVO2, GlideScope, and StorzDCI; (3) Airtraq significantly is associated with the reduced risk of LC compared with Macintosh and Pentax; (4) Airtraq ranks at first place for the improvement of FPS, followed by Miller and McGrath; (5) Canada ranks first for the reduction of TTI, followed by CMAC and Miller; and (6) KingVision ranks first for the reduction of LC, followed by Airtraq and Miller.

The Airtraq is a type of indirect laryngoscope with an exaggerated curative of the blade that is anatomically shaped^50^. It has mirrors and prisms that provide a wide-angle view of the airway during intubation, and the image is transmitted to a viewfinder^50^. Compared to the conventional laryngoscope, Airtraq does not need any alignment of oral, pharyngeal, and laryngeal axes for intubation and also has a quicker learning curve^30,31^. Studies in the adult population have demonstrated that the Airtraq laryngoscope facilitates faster and more accurate intubations^51^. Children have higher metabolisms and oxygen consumption than adults, and oxygen desaturation can more rapidly during intubation^35^; therefore, the FPS and TTI of intubation are especially important in pediatric patients. This meta-analysis has shown that Airtraq was superior to other video and conventional laryngoscopes in improving FPS, and the unique blade design and ease of learning might be contributing to the superiority. However, Airtraq involves a single-use, disposable blade, and its impact on the environment should be considered.

In included studies comparing other types of video laryngoscope with a conventional direct laryngoscope, authors found that proficiency is a main barrier to the FPS and TTI of video laryngoscope. Inal et al. stated that the primary reasons for the increased duration of TruviewEVO2 included less experience and lack of eye-hand coordination and practice^32^. Pangasa et al. also had similar findings^39^. Kim et al. also found that experience is needed to be skillful in the GlideScope to have comparable TTI with conventional intubation^34^. An earlier study suggested that a learning experience of 10–30 cases was required for proficiency in the use of GlideScope^52^. Sufficient training and practice of video laryngoscope should be implemented and evaluated when new devices are introduced to the clinical setting. Furthermore, the size and design of the blade should be taken into consideration when evaluating individual patients.

Yoo et al. and Kim et al. compared intubation methods in pediatric nasotracheal intubation^34,48^. Yoo et al. found that compared to the McGrath video laryngoscope and Pentax Airway Scope, the Macintosh laryngoscope has shorter TTI^48^, while Kim et al. demonstrated that GlideScope had similar performance as direct laryngoscope^34^. Further studies are needed to investigate the application of video laryngoscopes in pediatric nasotracheal intubation. Vadi et al. performed the comparison in manual in-line stabilization^43^. While the outcomes were similar among groups, the authors highlighted the importance of additional technical skills when performing video laryngoscopy^43^.

While some studies found that video laryngoscopes had comparable outcomes as the conventional ones, authors suggested that the optical system facilitates a clear image of the glottis which can be a useful option for tracheal intubation in patients with anticipated difficult airways^11,32, 40, 44^. Nonetheless, the included RCTs recruited pediatric patients scheduled for elective surgery, and generally excluded patients with difficult airways. Further studies are needed to evaluate whether different types of video laryngoscopes are suitable for children with difficult airways.

A recent pair-wise meta-analysis published in November 2020 investigated the comparative efficacy and safety of videolaryngoscopy versus direct laryngoscopy for intubation in children and suggested that videolaryngoscopy required a longer time to intubate and no difference was observed in FPS^1^. Unfortunately, this meta-analysis did not investigate the comparative efficacy and safety of individual videolaryngoscopy devices versus direct laryngoscopy devices or each other individually. In contrast to this meta-analysis, our network meta-analysis first compared individual videolaryngoscopy devices to direct laryngoscopy devices and also compared videolaryngoscopy devices to each other. Therefore, our results were informative in many clinical situations, as our network meta-analysis simultaneously determines the relative performance and ranking of different intubation devices. For example, Airtraq should be preferred for intubation if FPS was listed as the primary indicator of performance. Conversely, we should prioritize Canada if we decide to complete the intubation process within a limited time.

Although our network meta-analysis yielded some promising findings, we must also acknowledge that our network meta-analysis has several limitations: (1) By only including pediatric patients, variations of the results may be reduced. However, differences in ASA status, age, and weight may also confound our pooled results, as we did not perform sensitivity or subgroup analysis to eliminate the effect of these factors on the results; (2) We could not incorporate operator experience and patient characteristics into our analysis, as most studies did not report these outcomes. However, these factors are critical to clinical outcomes and may bias the pooled results significantly. Future studies should control for or report in detail on those confounding factors that play a crucial role in success and safety outcomes. (3) Cost-effectiveness is also an important factor in the choice of intubation. It is difficult to summarize the cost of intubation as the price of intubation equipment and procedures varies from hospital to hospital worldwide. (4)The methodological quality of the included studies varied, which also may affect the robustness of our pooled results; (4) small-study effect and publication bias were detected for TTI, which will reduce the reliability of this outcome to a certain extent; (5) direct comparison for some intubation devices is not available, so the pooled results were only calculated based on indirect comparisons, which also impair the robustness of pooled results; (6) some closed loops of TTI produced significant inconsistency, which will negatively influence our findings.

Despite these limitations above, our network meta-analysis has several advantages: (1) as far as we know, our network meta-analysis is the first attempt to comprehensively investigate the relative performance of different intubation devices in pediatric intubation; (2) our network meta-analysis ranks different intubation devices according to SUCRA value, which will aid in clinical decision-making; and (3) our network meta-analysis has also other strengths, such as the comprehensive search and use of the Cochrane risk of the bias assessment tool.

## Conclusion

For tracheal intubation in children, Airtraq may be the preferred intubation device because it significantly improves FPS, Canada may be the preferred option for the reduction of TTI, and Canada should be preferred for the significant reduction of LC. However, more studies are warranted to further validate our findings, as some intubation devices cannot be directly compared, and not all devices are included in individual outcomes.

### Statement

The work has been reported in line with PRISMA (Preferred Reporting Items for Systematic Reviews and Meta-Analyses) and AMSTAR (Assessing the methodological quality of systematic reviews) Guidelines.

### Supplementary Information

Supplementary Figures.

Supplementary Tables.

## Data Availability

All data generated or analysed during this study are included in this published article (and its supplementary information files).

## Abbreviations

ASA: American Society of Anaesthesiologists
TTI: Time to intubation
FPS: First-pass success
RCTs: Randomized comparative trials
