# Supplementary material for: Network meta-analysis of comparative efficacy and safety of intubation devices in children
Source: Sci Rep. 2023 Oct 30;13:18626. doi: 10.1038/s41598-023-45173-5 (PMC10616294; doi:10.1038/s41598-023-45173-5)
Supplement: Supplementary file 1 — Supplementary Figures. [file 41598_2023_45173_MOESM1_ESM.docx]

**Supplementary Materials**

**
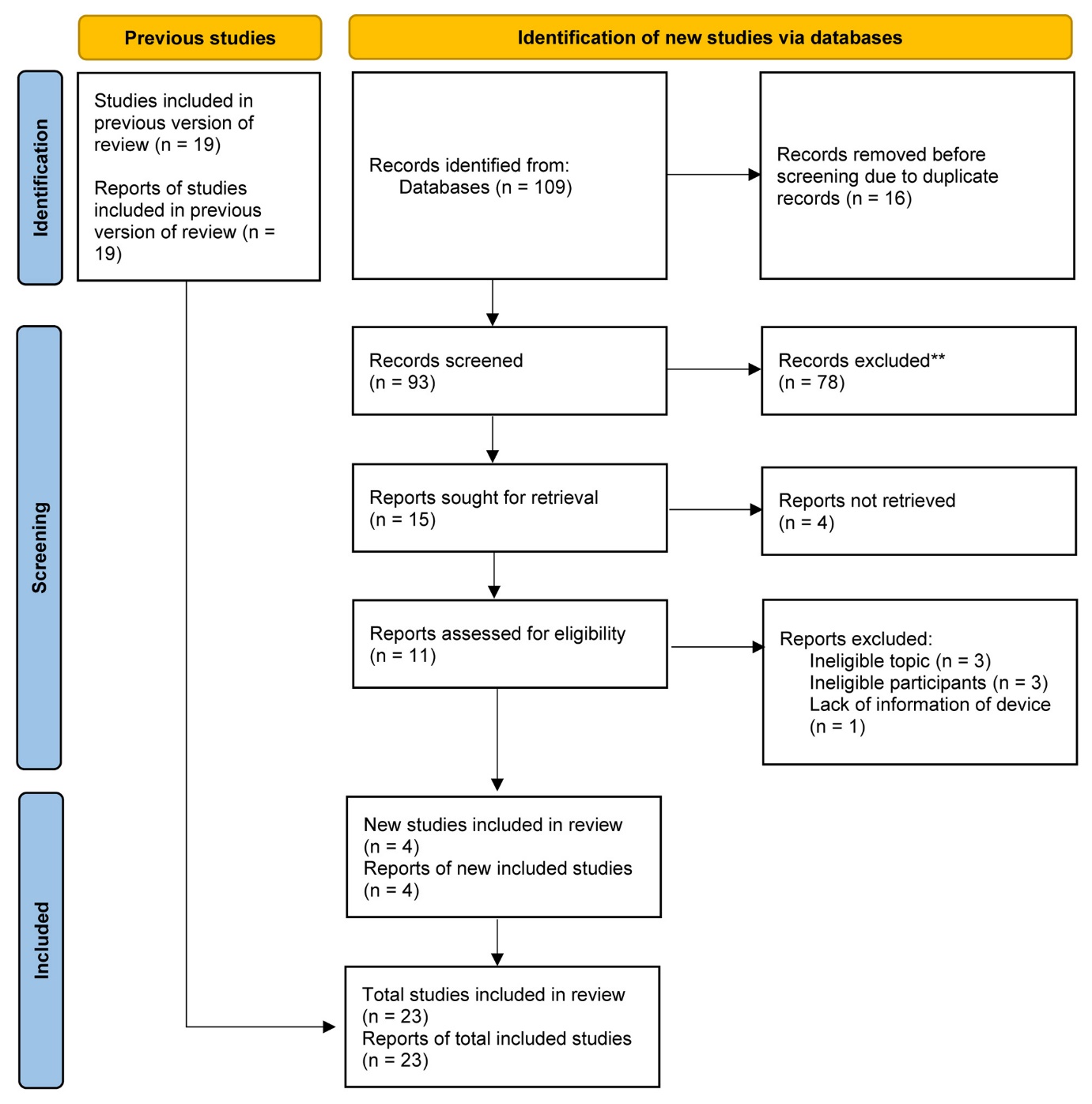
**

**Figure S1. PRISMA flow diagram.**

**
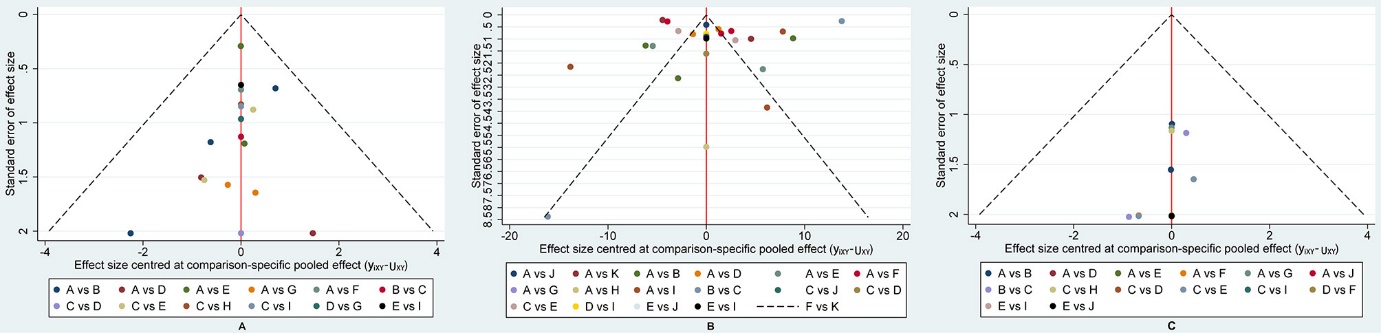
**

**Figure S2. Funnel plot for TTI for first-pass success (A), time to intubation (B), and local complications (C)**

**
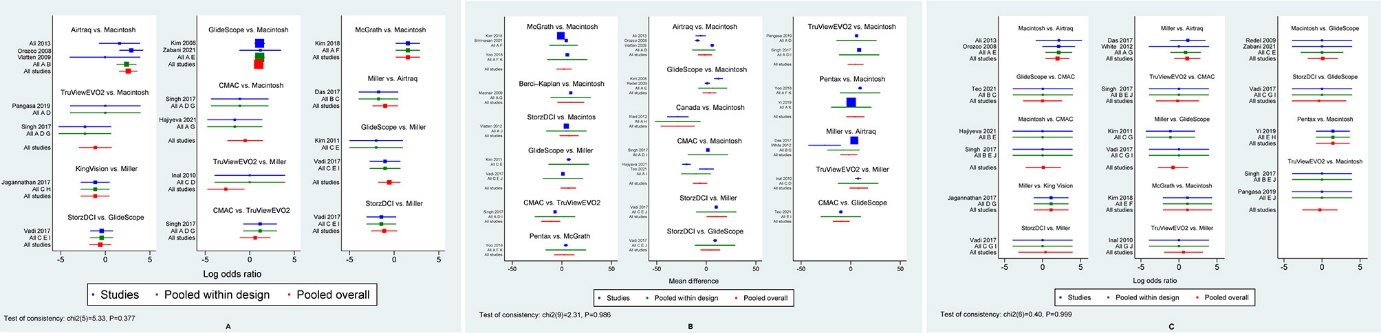
**

**Figure S3. Traditional pairwise analysis for first-pass success (A), time to intubation (B), and local complications (C).**

**
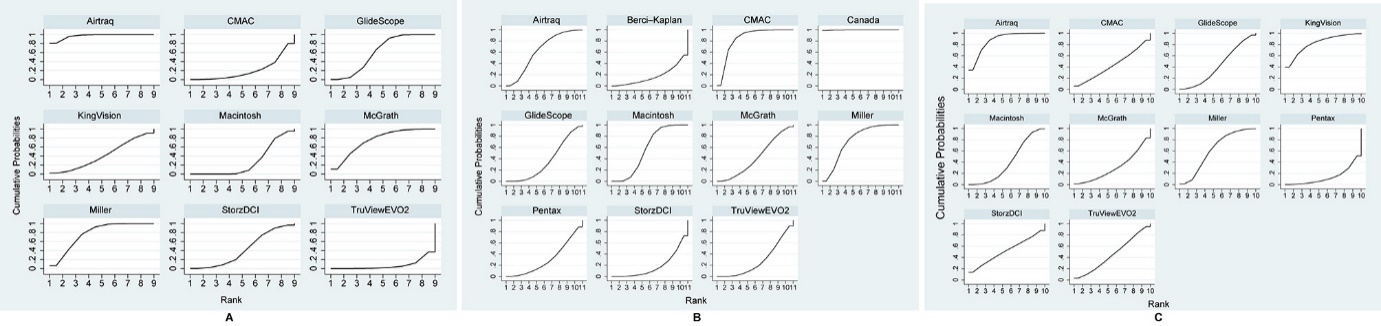
**

**Figure S4. SUCRA plots for for first-pass success (A), time to intubation (B), and local complications (C).**

**
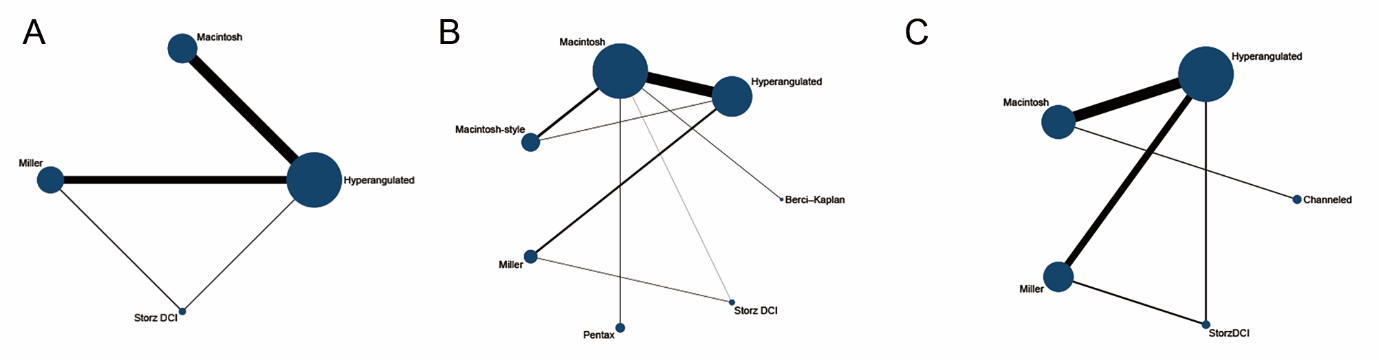
**

**Figure S5. Network plot for first-pass success (A), time to intubation (B), and local complications (C), Classified by type of laryngoscope**

**
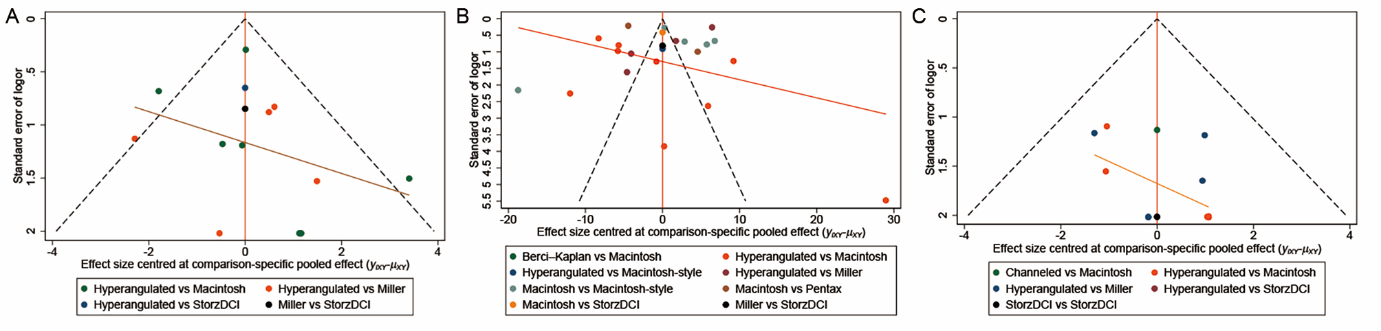
**

**Figure S6. Funnel plot for TTI for first-pass success (A), time to intubation (B), and local complications (C), Classified by type of laryngoscope**

**
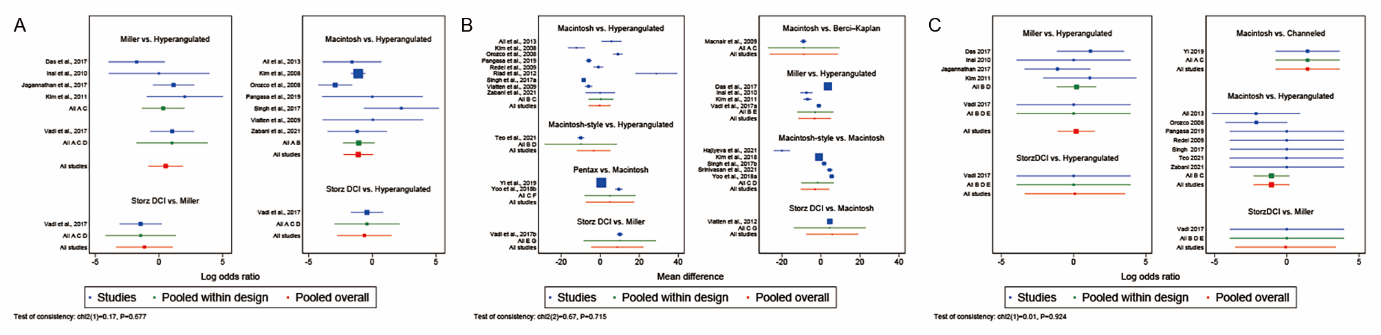
**

**Figure S7. Traditional pairwise analysis for first-pass success (A), time to intubation (B), and local complications (C), Classified by type of laryngoscope**

**
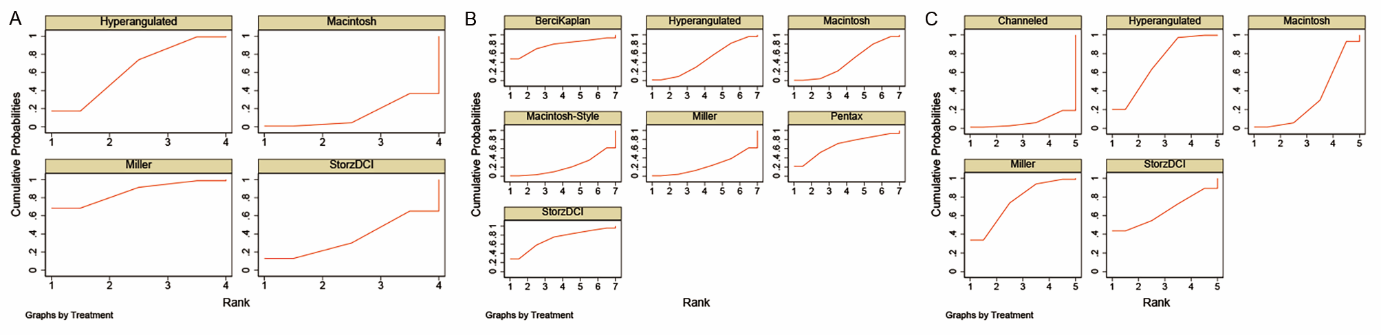
**

**Figure S8. SUCRA plots for for first-pass success (A), time to intubation (B), and local complications (C), Classified by type of laryngoscope**
