# Supplementary material for: Network meta-analysis of comparative efficacy and safety of intubation devices in children
Source: Sci Rep. 2023 Oct 30;13:18626. doi: 10.1038/s41598-023-45173-5 (PMC10616294; doi:10.1038/s41598-023-45173-5)
Supplement: Supplementary file 2 — Supplementary Tables. [file 41598_2023_45173_MOESM2_ESM.docx]

**Table S1. Search strategy in all database.**

**Pubmed**

| NO. | Query | Filters | Results |
| --- | --- | --- | --- |
| 7 | ((("Intubation, Intratracheal"[Mesh]) OR ((Intratracheal Intubation?) OR ((intubat* AND tracheal)))) AND ((((((((((video?laryngoscop*) OR ((video OR indrect) AND laryngoscop*))) OR (Storz videolaryngoscope)) OR (GlideScope)) OR (Truview)) OR (Pentax AWS)) OR (Airtraq)) OR (McGrath)) OR (C-MAC videolaryngoscope)) OR ('LMA CTrach' videolaryngoscope))) AND ((((((child[Title/Abstract]) OR (children[Title/Abstract])) OR (pediatric[Title/Abstract])) OR (infant*?[Title/Abstract])) OR (neonat*[Title/Abstract])) OR (newborn*[Title/Abstract])) | from 2020 - 2021 | 42 |
| 6 | ((("Intubation, Intratracheal"[Mesh]) OR ((Intratracheal Intubation?) OR ((intubat* AND tracheal)))) AND ((((((((((video?laryngoscop*) OR ((video OR indrect) AND laryngoscop*))) OR (Storz videolaryngoscope)) OR (GlideScope)) OR (Truview)) OR (Pentax AWS)) OR (Airtraq)) OR (McGrath)) OR (C-MAC videolaryngoscope)) OR ('LMA CTrach' videolaryngoscope))) AND ((((((child[Title/Abstract]) OR (children[Title/Abstract])) OR (pediatric[Title/Abstract])) OR (infant*?[Title/Abstract])) OR (neonat*[Title/Abstract])) OR (newborn*[Title/Abstract])) | | 298 |
| 5 | (((((child[Title/Abstract]) OR (children[Title/Abstract])) OR (pediatric[Title/Abstract])) OR (infant*?[Title/Abstract])) OR (neonat*[Title/Abstract])) OR (newborn*[Title/Abstract]) | | 2,074,674 |
| 4 | (((((((((video?laryngoscop*) OR ((video OR indrect) AND laryngoscop*))) OR (Storz videolaryngoscope)) OR (GlideScope)) OR (Truview)) OR (Pentax AWS)) OR (Airtraq)) OR (McGrath)) OR (C-MAC videolaryngoscope)) OR ('LMA CTrach' videolaryngoscope) | | 14,104 |
| 3 | ("Intubation, Intratracheal"[Mesh]) OR ((Intratracheal Intubation?) OR ((intubat* AND tracheal))) | | 47,882 |
| 2 | (Intratracheal Intubation?) OR ((intubat* AND tracheal)) | | 47,882 |
| 1 | "Intubation, Intratracheal"[Mesh] | | 41,003 |

**Embase**

| No. | Query | Results |
| --- | --- | --- |
| #5. | #4 AND (2021:py OR 2022:py) AND [embase]/lim | 59 |
| #4. | #1 AND #2 AND #3 | 588 |
| #3. | 'child'/exp OR child OR 'children'/exp OR  children OR 'full term infant'/exp OR 'full term  infant' OR (full AND term AND ('infant'/exp OR  infant)) OR 'human neonate'/exp OR 'human  neonate' OR (('human'/exp OR human) AND  ('neonate'/exp OR neonate)) OR 'human  newborn'/exp OR 'human newborn' OR (('human'/exp  OR human) AND ('newborn'/exp OR newborn)) OR  'neonatal animal'/exp OR 'neonatal animal' OR  (neonatal AND ('animal'/exp OR animal)) OR  'neonate'/exp OR neonate OR 'neonate animal'/exp  OR 'neonate animal' OR (('neonate'/exp OR  neonate) AND ('animal'/exp OR animal)) OR  'neonatus'/exp OR neonatus OR 'newborn'/exp OR  newborn OR 'newborn animal'/exp OR 'newborn  animal' OR (('newborn'/exp OR newborn) AND  ('animal'/exp OR animal)) OR 'newborn baby'/exp  OR 'newborn baby' OR (('newborn'/exp OR newborn)  AND ('baby'/exp OR baby)) OR 'newborn child'/exp  OR 'newborn child' OR (('newborn'/exp OR newborn)  AND ('child'/exp OR child)) OR 'newborn  infant'/exp OR 'newborn infant' OR  (('newborn'/exp OR newborn) AND ('infant'/exp OR  infant)) OR 'newly born baby'/exp OR 'newly born  baby' OR (newly AND born AND ('baby'/exp OR  baby)) OR 'newly born child'/exp OR 'newly born  child' OR (newly AND born AND ('child'/exp OR  child)) OR 'newly born infant'/exp OR 'newly born  infant' OR (newly AND born AND ('infant'/exp OR  infant)) | 4,306,645 |
| #2. | 'a.p. advance'/exp OR 'a.p. advance' OR (a.p. AND  ('advance'/exp OR advance)) OR 'aws s100l'/exp OR  'aws s100l' OR 'aws s200'/exp OR 'aws s200' OR  'airway scope'/exp OR 'airway scope' OR  (('airway'/exp OR airway) AND ('scope'/exp OR  scope)) OR 'airway scope aws-s200'/exp OR 'airway  scope aws-s200' OR (('airway'/exp OR airway) AND  ('scope'/exp OR scope) AND ('aws s200'/exp OR  'aws s200')) OR 'c mac'/exp OR 'c mac' OR  'endostrob'/exp OR endostrob OR 'glidescope'/exp  OR glidescope OR 'glidescope avl'/exp OR  'glidescope avl' OR (('glidescope'/exp OR  glidescope) AND avl) OR 'glidescope ranger'/exp  OR 'glidescope ranger' OR (('glidescope'/exp OR  glidescope) AND ('ranger'/exp OR ranger)) OR  'glidescope titanium'/exp OR 'glidescope  titanium' OR (('glidescope'/exp OR glidescope)  AND ('titanium'/exp OR titanium)) OR 'king  vision'/exp OR 'king vision' OR (king AND  ('vision'/exp OR vision)) OR 'mcgrath'/exp OR  mcgrath OR 'mcgrath mac'/exp OR 'mcgrath mac' OR  (('mcgrath'/exp OR mcgrath) AND mac) OR 'storz  dci'/exp OR 'storz dci' OR (('storz'/exp OR  storz) AND ('dci'/exp OR dci)) OR 'truview'/exp  OR truview OR 'truview pcd'/exp OR 'truview pcd'  OR (('truview'/exp OR truview) AND ('pcd'/exp OR  pcd)) OR 'vlp 100'/exp OR 'vlp 100' OR 'video  laryngoscope'/exp OR 'video laryngoscope' OR  (('video'/exp OR video) AND ('laryngoscope'/exp  OR laryngoscope)) OR 'videolaryngoscope'/exp OR  videolaryngoscope | 25,121 |
| #1. | endotracheal AND ('intubation'/exp OR intubation) OR 'intratracheal intubation'/exp OR  'intratracheal intubation' OR (intratracheal AND  ('intubation'/exp OR intubation)) OR 'orotracheal  intubation'/exp OR 'orotracheal intubation' OR  (orotracheal AND ('intubation'/exp OR  intubation)) OR 'trachea intubation'/exp OR  'trachea intubation' OR (('trachea'/exp OR  trachea) AND ('intubation'/exp OR intubation)) OR  'tracheal intubation'/exp OR 'tracheal intubation' OR (tracheal AND ('intubation'/exp OR  intubation)) OR 'endotracheal intubation'/exp OR  'endotracheal intubation' | 70,156 |

**Cochrane Central Registry of Controlled Trials**

| ID | Search | Hits |
| --- | --- | --- |
| #1 | MeSH descriptor: [Intubation, Intratracheal] explode all trees | 4589 |
| #2 | (Intratracheal Intubation?):ti,ab,kw | 4142 |
| #3 | #1 or #2 | 4752 |
| #4 | (videolaryngoscope):ti,ab,kw OR (Storz videolaryngoscope):ti,ab,kw OR (GlideScope):ti,ab,kw OR (Truview):ti,ab,kw OR (Pentax AWS):ti,ab,kw | 1175 |
| #5 | (Airtraq):ti,ab,kw OR (McGrath):ti,ab,kw OR (C-MAC videolaryngoscope):ti,ab,kw OR ('LMA CTrach' videolaryngoscope):ti,ab,kw OR ((video OR indrect) AND laryngoscop*):ti,ab,kw | 1510 |
| #6 | #4 or #5 | 1855 |
| #7 | (Child):ti,ab,kw OR (Children):ti,ab,kw OR (pediatric):ti,ab,kw OR (newborn):ti,ab,kw OR (neonate):ti,ab,kw | 184886 |
| #8 | (infant):ti,ab,kw | 53048 |
| #9 | #7 or #8 | 193904 |
| #10 | #3 and #6 and #9 | 78 |

**Table S2. Local inconsistency examination with node-split method**

| Loop | IF (95% CI) | z_value | p_value | Loop_Heterog_tau2 |
| --- | --- | --- | --- | --- |
| FPS | | | | |
| A-C-D-E | 3.854 (0.00,8.73) | 1.549 | 0.121 | 0.000 |
| A-B-C-D | 1.972 (0.00,7.41) | 0.711 | 0.477 | 0.183 |
| A-B-C-E | 1.767 (0.00,4.71) | 1.176 | 0.240 | 0.000 |
| A-D-G | 1.377 (0.00,5.70) | 0.625 | 0.532 | 0.000 |
| C-E-I | 0.997 (0.00,4.65) | 0.535 | 0.593 | 0.000 |
| TTI | | | | |
| A-D-I | 15.902 (11.80,20.00) | 7.602 | 0.000 | 0.000 |
| A-B-C-E | 10.720 (0.00,38.11) | 0.767 | 0.443 | 4.207 |
| A-E-J | 8.167 (5.13,11.20) | 5.277 | 0.000 | 0.000 |
| C-D-E-I | 7.772 (3.68,11.86) | 3.724 | 0.000 | 0.000 |
| A-B-C-D | 6.442 (0.00,36.42) | 0.535 | 0.593 | 0.000 |
| C-E-J | 5.800 (2.56,9.04) | 3.506 | 0.000 | 0.000 |
| A-C-D-J | 5.638 (1.90,9.38) | 2.952 | 0.003 | 5.682 |
| A-B-C-J | 4.845 (0.00,46.49) | 0.228 | 0.820 | 1.272 |
| A-E-I | 2.724 (0.00,33.53) | 0.173 | 0.862 | 2.214 |
| A-F-K | 2.708 (0.68,4.74) | 2.617 | 0.009 | 0.000 |
| A-C-D-E | 2.286 (0.00,18.19) | 0.282 | 0.778 | 2.226 |
| LC | | | | |
| A-B-C-D | 1.249 (0.00,6.78) | 0.443 | 0.658 | 0.000 |
| C-E-I | 1.124 (0.00,7.58) | 0.341 | 0.733 | 0.000 |
| A-D-F | 1.122 (0.00,6.93) | 0.379 | 0.705 | 0.000 |
| A-C-D-E | 0.673 (0.00,6.80) | 0.215 | 0.829 | 0.000 |
| A-B-C-E | 0.575 (0.00,5.18) | 0.245 | 0.806 | 0.000 |
| A-E-J | 0.030 (0.00,6.28) | 0.010 | 0.992 | 0.000 |

IF, inconsistent factor; CI, confidence interval; FPS, first-pass success; TTI, time to intubation; LC, local complications. For FPS, A, B, C, D, E, G, I represent Macintosh, Airtraq, Miller, TruViewEVO2, GlideScope, CMAC, StorzDCI, respectively. For TTI, A, B, C, D, E, F, I, J, K represent Macintosh, Airtraq, Miller, TruViewEVO2, GlideScope, McGrath, CMAC, StorzDCI, Pentax, respectively. For LC, A, B, C, D, E, F, I, J represent Macintosh, Airtraq, Miller, TruViewEVO2, GlideScope, McGrath, StorzDCI, CMAC, respectively.

NOTE: Table S2 provide information on the difference between the effect estimates based on direct and indirect evidence and indicate whether this difference is significant or not

**Table S3. League table of FPS, TTI, and LC.**

1. League table of TTI

| Canada | 5.53e+09 (44.17,6.93e+17) | 1.22e+11 (731.62,2.02e+19) | 8.86e+11 (7327.37,1.07e+20) | 3.09e+12 (102742.57,9.31e+19) | 3.01e+13 (199492.19,4.55e+21) | 7.98e+13 (689898.44,9.23e+21) | 3.33e+14 (1.14e+06,9.73e+22) | 4.00e+14 (2.26e+06,7.06e+22) | 4.58e+15 (1.28e+07,1.64e+24) | 2.51e+16 (7.58e+06,8.29e+25) |
| --- | --- | --- | --- | --- | --- | --- | --- | --- | --- | --- |
| 0.00 (0.00,0.02) | CMAC | 21.97 (0.00,362439.38) | 160.23 (0.01,2.75e+06) | 559.07 (0.44,712822.56) | 5444.81 (0.16,1.89e+08) | 14424.33 (2.93,7.09e+07) | 60210.09 (0.55,6.58e+09) | 72256.03 (5.72,9.13e+08) | 828660.13 (9.41,7.30e+10) | 4.53e+06 (0.99,2.06e+13) |
| 0.00 (0.00,0.00) | 0.05 (0.00,750.59) | Miller | 7.29 (0.00,38335.57) | 25.44 (0.01,65822.44) | 247.78 (0.00,1.41e+07) | 656.42 (0.27,1.62e+06) | 2740.01 (0.02,4.70e+08) | 3288.19 (0.31,3.53e+07) | 37710.25 (1.39,1.02e+09) | 206157.53 (0.03,1.32e+12) |
| 0.00 (0.00,0.00) | 0.01 (0.00,107.29) | 0.14 (0.00,721.00) | Airtraq | 3.49 (0.00,4061.70) | 33.98 (0.00,1.11e+06) | 90.02 (0.01,798685.88) | 375.77 (0.00,3.87e+07) | 450.95 (0.02,1.08e+07) | 5171.63 (0.07,3.88e+08) | 28272.70 (0.01,1.23e+11) |
| 0.00 (0.00,0.00) | 0.00 (0.00,2.28) | 0.04 (0.00,101.69) | 0.29 (0.00,333.64) | Macintosh | 9.74 (0.00,19950.16) | 25.80 (0.02,26660.08) | 107.70 (0.01,998901.19) | 129.24 (0.04,387725.72) | 1482.21 (0.10,2.11e+07) | 8103.08 (0.01,6.29e+09) |
| 0.00 (0.00,0.00) | 0.00 (0.00,6.37) | 0.00 (0.00,229.93) | 0.03 (0.00,958.01) | 0.10 (0.00,210.34) | McGrath | 2.65 (0.00,79628.83) | 11.06 (0.00,372503.97) | 13.27 (0.00,840737.31) | 152.19 (0.00,3.12e+07) | 832.02 (0.00,4.75e+09) |
| 0.00 (0.00,0.00) | 0.00 (0.00,0.34) | 0.00 (0.00,3.77) | 0.01 (0.00,98.56) | 0.04 (0.00,40.05) | 0.38 (0.00,11346.05) | GlideScope | 4.17 (0.00,401329.59) | 5.01 (0.00,75617.95) | 57.45 (0.00,1.18e+06) | 314.07 (0.00,1.30e+09) |
| 0.00 (0.00,0.00) | 0.00 (0.00,1.81) | 0.00 (0.00,62.66) | 0.00 (0.00,274.17) | 0.01 (0.00,86.12) | 0.09 (0.00,3046.20) | 0.24 (0.00,23033.18) | Pentax | 1.20 (0.00,226510.23) | 13.76 (0.00,7.62e+06) | 75.24 (0.00,9.50e+08) |
| 0.00 (0.00,0.00) | 0.00 (0.00,0.17) | 0.00 (0.00,3.26) | 0.00 (0.00,53.06) | 0.01 (0.00,23.21) | 0.08 (0.00,4773.95) | 0.20 (0.00,3013.47) | 0.83 (0.00,157281.53) | TruViewEVO2 | 11.47 (0.00,1.49e+06) | 62.70 (0.00,4.33e+08) |
| 0.00 (0.00,0.00) | 0.00 (0.00,0.11) | 0.00 (0.00,0.72) | 0.00 (0.00,14.50) | 0.00 (0.00,9.58) | 0.01 (0.00,1345.35) | 0.02 (0.00,356.18) | 0.07 (0.00,40248.69) | 0.09 (0.00,11336.38) | StorzDCI | 5.47 (0.00,8.79e+07) |
| 0.00 (0.00,0.00) | 0.00 (0.00,1.01) | 0.00 (0.00,31.12) | 0.00 (0.00,154.43) | 0.00 (0.00,95.77) | 0.00 (0.00,6867.43) | 0.00 (0.00,13162.27) | 0.01 (0.00,167869.14) | 0.02 (0.00,110258.02) | 0.18 (0.00,2.94e+06) | Berci–Kaplan |

1. League table of FPS

| Airtraq | 0.37 (0.09,1.52) | 0.35 (0.06,1.94) | 0.21 (0.07,0.65) | 0.12 (0.02,0.59) | 0.12 (0.01,1.02) | 0.08 (0.03,0.21) | 0.05 (0.00,0.42) | 0.02 (0.00,0.20) |
| --- | --- | --- | --- | --- | --- | --- | --- | --- |
| 2.73 (0.66,11.39) | Miller | 0.97 (0.15,6.27) | 0.58 (0.17,2.00) | 0.33 (0.08,1.39) | 0.32 (0.06,1.62) | 0.21 (0.06,0.76) | 0.13 (0.01,1.22) | 0.07 (0.01,0.54) |
| 2.83 (0.51,15.55) | 1.03 (0.16,6.72) | McGrath | 0.60 (0.14,2.59) | 0.34 (0.05,2.26) | 0.33 (0.03,3.94) | 0.22 (0.06,0.85) | 0.13 (0.01,1.45) | 0.07 (0.01,0.69) |
| 4.73 (1.54,14.58) | 1.73 (0.50,5.99) | 1.67 (0.39,7.24) | GlideScope | 0.57 (0.17,1.93) | 0.55 (0.07,4.28) | 0.36 (0.21,0.62) | 0.22 (0.03,1.69) | 0.12 (0.02,0.78) |
| 8.31 (1.70,40.62) | 3.04 (0.72,12.86) | 2.94 (0.44,19.48) | 1.76 (0.52,5.95) | StorzDCI | 0.97 (0.11,8.54) | 0.64 (0.17,2.37) | 0.38 (0.04,4.00) | 0.21 (0.02,1.86) |
| 8.55 (0.98,74.35) | 3.13 (0.62,15.89) | 3.02 (0.25,36.02) | 1.81 (0.23,13.97) | 1.03 (0.12,9.03) | KingVision | 0.66 (0.08,5.20) | 0.39 (0.02,6.42) | 0.21 (0.02,2.98) |
| 13.05 (4.68,36.38) | 4.77 (1.32,17.22) | 4.61 (1.18,17.99) | 2.76 (1.60,4.75) | 1.57 (0.42,5.84) | 1.53 (0.19,12.10) | Macintosh | 0.60 (0.08,4.40) | 0.33 (0.05,2.04) |
| 21.80 (2.36,201.20) | 7.97 (0.82,77.52) | 7.71 (0.69,86.20) | 4.61 (0.59,35.94) | 2.62 (0.25,27.52) | 2.55 (0.16,41.72) | 1.67 (0.23,12.28) | CMAC | 0.54 (0.10,3.03) |
| 40.08 (5.07,316.73) | 14.66 (1.84,117.04) | 14.17 (1.44,138.96) | 8.47 (1.28,56.08) | 4.82 (0.54,43.26) | 4.69 (0.34,65.51) | 3.07 (0.49,19.21) | 1.84 (0.33,10.23) | TruViewEVO2 |

1. League table of FPS, classified by type of laryngoscope

| Hyperangulated | 0.33 (0.10,1.06) | 1.68 (0.43,6.53) | 0.53 (0.06,4.59) |
| --- | --- | --- | --- |
| 3.02 (0.94,9.67) | Macintosh | 5.07 (0.84,30.67) | 1.61 (0.14,18.79) |
| 0.59 (0.15,2.31) | 0.20 (0.03,1.19) | Miller | 0.32 (0.03,2.92) |
| 1.87 (0.22,16.09) | 0.62 (0.05,7.24) | 3.15 (0.34,28.98) | StorzDCI |

1. League table of TTI, classified by type of laryngoscope

| BerciKaplan | 0.00 (0.00,19727.38) | 0.00 (0.00,5713.15) | 0.00 (0.00,1172.75) | 0.00 (0.00,3928.77) | 0.02 (0.00,4.44e+07) | 0.05 (0.00,1.73e+08) |
| --- | --- | --- | --- | --- | --- | --- |
| 4796.92 (0.00,4.54e+11) | Hyperangulated | 0.70 (0.00,203.63) | 0.03 (0.00,188.43) | 0.04 (0.00,166.82) | 101.35 (0.00,8.98e+07) | 233.04 (0.00,1.71e+08) |
| 6866.88 (0.00,2.69e+11) | 1.43 (0.00,417.30) | Macintosh | 0.05 (0.00,70.54) | 0.06 (0.00,950.48) | 145.08 (0.00,3.75e+07) | 333.60 (0.00,2.13e+08) |
| 142319.72 (0.00,2.38e+13) | 29.67 (0.01,165862.00) | 20.73 (0.01,30300.14) | **Macintoshstyle** | 1.16 (0.00,148763.11) | 3006.95 (0.00,5.60e+09) | 6913.98 (0.00,2.41e+10) |
| 122382.97 (0.00,5.88e+13) | 25.51 (0.01,108587.04) | 17.82 (0.00,301902.06) | 0.86 (0.00,110003.65) | Miller | 2585.72 (0.00,1.91e+10) | 5945.44 (0.01,3.84e+09) |
| 47.33 (0.00,9.95e+10) | 0.01 (0.00,8747.20) | 0.01 (0.00,1782.61) | 0.00 (0.00,619.53) | 0.00 (0.00,2860.74) | Pentax | 2.30 (0.00,1.99e+08) |
| 20.58 (0.00,7.33e+10) | 0.00 (0.00,3145.88) | 0.00 (0.00,1914.20) | 0.00 (0.00,504.99) | 0.00 (0.00,108.74) | 0.43 (0.00,3.76e+07) | StorzDCI |

1. League table of LC, classified by type of laryngoscope

| Channeled | 12.21 (0.96,155.35) | 4.19 (0.46,38.45) | 14.65 (0.85,253.59) | 13.38 (0.18,997.35) |
| --- | --- | --- | --- | --- |
| 0.08 (0.01,1.04) | Hyperangulated | 0.34 (0.10,1.19) | 1.20 (0.33,4.36) | 1.10 (0.03,35.61) |
| 0.24 (0.03,2.19) | 2.92 (0.84,10.13) | Macintosh | 3.50 (0.58,21.01) | 3.20 (0.08,128.94) |
| 0.07 (0.00,1.18) | 0.83 (0.23,3.03) | 0.29 (0.05,1.72) | Miller | 0.91 (0.03,29.69) |
| 0.07 (0.00,5.57) | 0.91 (0.03,29.69) | 0.31 (0.01,12.63) | 1.10 (0.03,35.61) | StorzDCI |
